# NMR Profiling of Metabolites in Larval and Juvenile Blue Mussels (*Mytilus edulis*) under Ambient and Low Salinity Conditions

**DOI:** 10.3390/metabo7030033

**Published:** 2017-07-06

**Authors:** Melissa A. May, Karl D. Bishop, Paul D. Rawson

**Affiliations:** 1 5751 Murray Hall, School of Marine Sciences, University of Maine, Orono, ME 04469, USA; prawson@maine.edu; 2 Biochemistry Department, Husson University, 1 College Circle, Bangor, ME 04401, USA; bishopk@husson.edu

**Keywords:** blue mussels (*Mytilus edulis*), NMR profiling, metabolomics, osmotic stress

## Abstract

Blue mussels (*Mytilus edulis*) are ecologically and economically important marine invertebrates whose populations are at risk from climate change-associated variation in their environment, such as decreased coastal salinity. Blue mussels are osmoconfomers and use components of the metabolome (free amino acids) to help maintain osmotic balance and cellular function during low salinity exposure. However, little is known about the capacity of blue mussels during the planktonic larval stages to regulate metabolites during osmotic stress. Metabolite studies in species such as blue mussels can help improve our understanding of the species’ physiology, as well as their capacity to respond to environmental stress. We used 1D ^1^H nuclear magnetic resonance (NMR) and 2D total correlation spectroscopy (TOCSY) experiments to describe baseline metabolite pools in larval (veliger and pediveliger stages) and juvenile blue mussels (gill, mantle, and adductor tissues) under ambient conditions and to quantify changes in the abundance of common osmolytes in these stages during low salinity exposure. We found evidence for stage- and tissue-specific differences in the baseline metabolic profiles of blue mussels, which reflect variation in the function and morphology of each larval stage or tissue type of juveniles. These differences impacted the utilization of osmolytes during low salinity exposure, likely stemming from innate physiological variation. This study highlights the importance of foundational metabolomic studies that include multiple tissue types and developmental stages to adequately evaluate organismal responses to stress and better place these findings in a broader physiological context.

## 1. Introduction

The blue mussel (*Mytilus edulis*) is an important marine species that is commonly found in intertidal and subtidal habitats of the temperate and sub-boreal regions of the North Atlantic. Ecologically, *M. edulis* is considered a foundational species, as it provides habitat and structure within coastal ecosystems [1]. Additionally, mussels are of importance because they are commercially fished and cultured and commonly used in environmental monitoring programs [2,3,4,5,6,7]. Thus, any factor that threatens the health of mussel populations also poses a risk for other species within the community and impacts the quality of mussels as food sources or as bioindicators. Given the increasing pace of environmental change, there is substantial interest in the physiological tolerances of *M. edulis* and the effects of environmental stress on the persistence of blue mussel populations e.g., [8,9,10]. 

To adequately understand the capacity for species to respond to environmental stress, there is a critical need for foundational metabolomic studies that provide the baselines for future comparisons. While previous studies have presented data on the metabolomic signatures of unstressed mussels [8,11,12], these studies have often focused on a single tissue or did not address the naturally occurring variation in the metabolome among tissue types. In addition, mussels have a complex life cycle that includes a protracted planktonic period of larval development so that studies on adult mussels are only representative of the physiological state of post-metamorphic, benthic stages. The energetic demands of larvae differ from those of post-metamorphic mussels, as they must obtain adequate energy stores to reach competency and undergo metamorphosis [13,14]. Thus, a comprehensive metabolomic study of species with complex life-histories, like *M. edulis*, must also account for developmental changes in physiology. 

Ontogenetic variation in the metabolome may also impact the ability of mussels to respond to osmotic stress. Adult mussels selectively retain free amino acids (FAA), which function as osmolytes, within their metabolite pools as a means of intracellular osmotic regulation [15,16,17,18]. Variation in the composition and utilization of FAA pools in response to osmotic stress have been widely studied in many marine bivalves e.g., [19,20,21,22,23]. However, there have been no studies to date that have looked broadly at the metabolic changes that occur across developmental stages or across tissues in juvenile mussels when subjected to osmotic stress. Bivalve larvae differ from juveniles in their uptake and metabolism of FAAs [24,25], so it is likely that the larval response to changes in environmental salinity differs from that of juvenile and adult mussels. 

Nuclear magnetic resonance spectroscopy (NMR) is a commonly used tool for studying the metabolic profiles of bivalve molluscs [7,8,9,11,12,26,27,28,29]. NMR rapidly generates extensive, quantitative metabolomic data, and can be used to detect the presence of solutes in low concentrations or in small samples. We used NMR spectroscopy to examine the baseline composition of the metabolome, with attention to the composition of the FAA pools, in larval (veliger and pediveliger stages) and juvenile blue mussels reared under control conditions (13.5 °C, 32 ppt). Additionally, we monitored changes in the FAA pools of hypoosmotically challenged larval and juvenile mussels to assess how ontogenetic variation in the metabolome affects the ability of mussels to respond. This study provides important physiological baseline data on *M. edulis* that are integral to understanding the cellular changes that occur during hypoosmotic stress. 

## 2. Results

### 2.1. Baseline Metabolic Profiles

**Figure 1:**
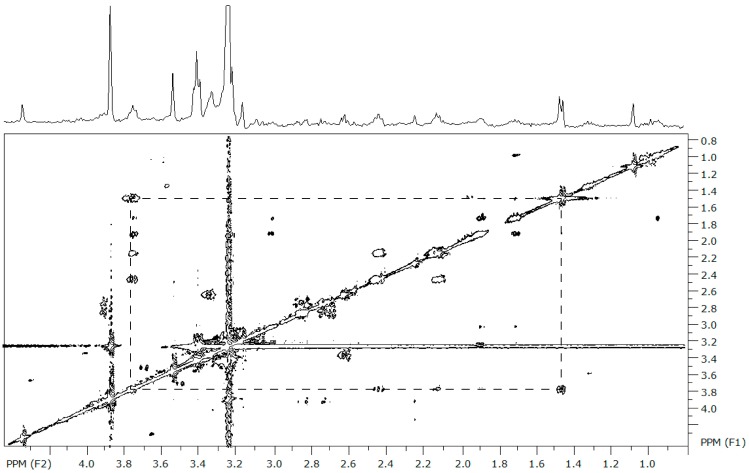
The representative 2D total correlation spectroscopy (TOCSY) spectrum from *Mytilus edulis* over 0–5 ppm. The spectrum is referenced to the chemical shift of TSP and the D_2_O peak was removed. The box on the 2D plot connects the cross-peaks contributed by the resonances of the hydrogen atoms within alanine, where there is a doublet at 1.46 ppm and a triplet at 3.77 ppm. These coupling patterns are used to verify the identity of the compounds in Table 1; the complete list of all coupling partners generated from the TOCSY experiments is provided in Table S1.

We detected 99 distinct metabolic signatures using NMR on extracts from two larval stages and from the gill, mantle, and adductor tissues of juvenile *Mytilus edulis*. We confirmed the identity of 16 of these 99 metabolites (Table 1) using information obtained from the 2D total correlation spectroscopy (TOCSY) data (Figure 1). Interestingly, only 9 of the 16 identified metabolites were common to both larval stages and all three tissues of the juvenile mussels: alanine, aspartate, betaine, glycine, homarine, hypotaurine, isoleucine, taurine, and threonine (Table 1, metabolites 1–9). Arginine, β-alanine, glutamate, glutamine, leucine, lysine, as well as four unknown metabolites, were found in larval and juvenile mussels, but were not always detected in both larval stages or samples from all three tissues of the juveniles (metabolites 10–19, Table 1). Among these metabolites there are amino acids and amino acid derivatives that appear to be the major constituents of the M. edulis intracellular FAA pool, regardless of developmental stage. 

**Figure 2:**
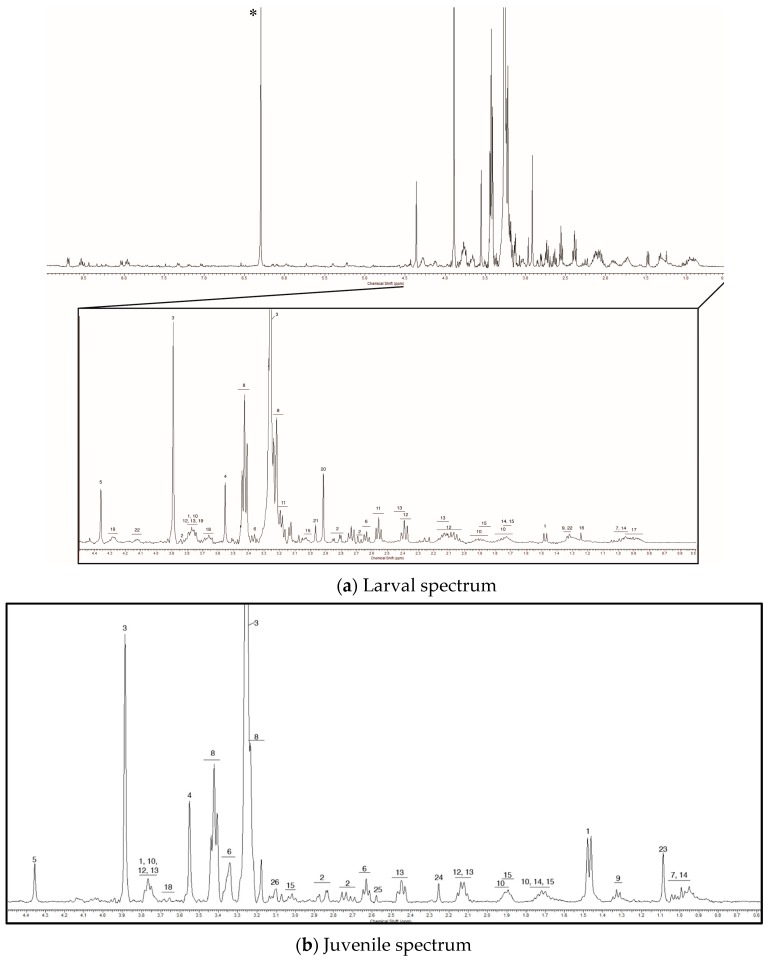
A representative 1D ^1^H NMR spectra for larval (**a**) veliger and juvenile (**b**) mantle tissue mussels are shown over the 0–9 ppm range, with a focus on the 0–4.5 ppm range where the chemical shifts for most of the metabolites we detected are found. The numbers above each peak correspond to the metabolites listed in Table 1. The spectra are referenced to the chemical shift of trimethylsilylpropanoic acid (TSP) (0 ppm). The asterisk in panel a marks the chemical shift for the maleic acid spike (6.29 ppm) that was used for relative quantification.

We found several metabolites, however, whose presence or relative concentration varied across developmental stages. Two prominent peaks at 2.92 and 2.96 ppm were present in the veliger and pediveliger samples, but were not detected in any of the juvenile samples (Figure 2a). These compounds likely comprise a large portion of the metabolite pool in larvae, but could not be identified with information present in the literature or online databases. Lactic acid also appeared to be specific to larvae, although, the chemical shift of lactic acid overlaps with that of threonine (and other metabolites at 1.32 ppm). Thus, it is possible that lactic acid was present in the juvenile samples, but that the concentrations were too low to see the long-range coupling protons at 4.12 ppm. In addition to the larval-specific metabolites, we observed four unknown metabolites specific to juveniles at 1.09, 2.25, 2.56 and 3.11 ppm (Figure 2b). The intensity of the peaks was higher in some tissues, indicating that there is tissue-specific variation in the concentration of these metabolites in post-metamorphic mussels.

While we observed the presence of stage-specific and tissue-specific metabolites, we could not determine the identity of most of the metabolites we detected (Table S1). There were 13 metabolites found only in veligers and an additional 13 found only in pediveligers. In juvenile mussels, we observed 15 metabolites that were unique to the gill, 9 that were found only in the mantle tissue, and 12 in the adductor muscle. Surprisingly, we did not observe β-alanine or another metabolite, identified as “Unknown #2” by Tikunov et al. [26], in the mantle of juvenile M. edulis even though these metabolites were present in our other samples. Similarly, unknown metabolite #18 (Table 1) was absent from the adductor muscle, but present in the gill and mantle tissue, as well as in veliger and pediveliger larvae. Other metabolites were detected in our pediveliger samples and samples from juvenile tissues, but not in profiles from veliger larvae (Table S1). In contrast, we did not detect any metabolites common to veligers and juveniles but missing in the pediveliger profiles.

**Figure 3:**
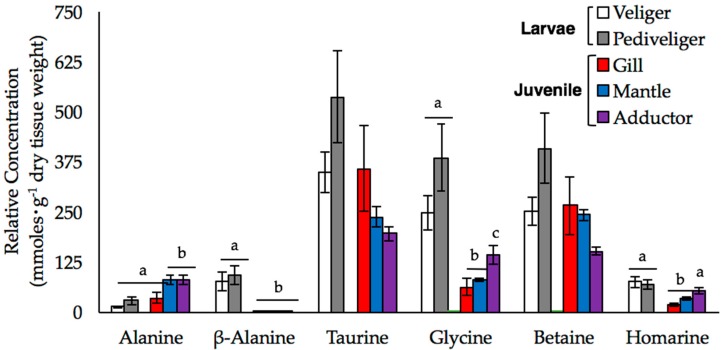
The mean relative concentrations of alanine, β-alanine, taurine, glycine, betaine, and homarine (±SE) are shown for larval and juvenile mussels. Larval samples were analyzed at both the veliger (white bars; *n* = 3) and pediveliger (gray bars; *n* = 4) stages, while data from the gill (black bars), mantle (red bars), and adductor muscle (blue bars) were obtained from the tissues of individual juveniles (*n* = 5). Letters denote significant differences between the stages or tissues (at an experiment-wide α = 0.05).

We used our 1D NMR data to investigate changes in the relative concentrations of six metabolites, alanine, β-alanine, betaine, glycine, homarine, and taurine, in more detail. These compounds play an important role in the FAA pool of mussels [19,20,21,22,23]. We detected significant multivariate main effect of sample type (tissue type or stage) on the concentration of these six amino acids (Wilk’s λ = 0.002, F_24,40.07_ = 9.669, *p* < 0.001, power = 0.999). Examination of univariate effects of sample type determined there were significant differences in amino acid concentration among tissue types or stages for alanine (F_4,17_ = 7.11, *p* = 0.001), β-alanine (F_4,17_ = 15.213, *p* < 0.001), glycine (F_4,17_ = 11.25, *p* < 0.001), and homarine (F_4,17_ = 9.082, *p* < 0.001) after adjusting *p*-value for the multiple one-way ANOVAs (critical value *p* < 0.008). Overall, taurine, betaine, and glycine were the most abundant FAAs in all samples (Figure 3).

In juveniles, the FAA pools were dominated by taurine and betaine, and to a lesser extent, glycine and alanine, while in larvae, glycine was just as abundant as taurine and betaine (Figure 3). The composition of the FAA pools of veliger and pediveliger larvae were similar; although the concentrations of all six FAAs, and taurine and betaine, in particular, tended to be higher in the pediveligers than in the veligers and juvenile tissues, these differences were not statistically significant. We observed both stage- and tissue-specific differences in the concentrations of glycine and homarine, which were higher in the larval stages and in the adductor muscle than in the gill and mantle tissues of juveniles. The accumulation of β-alanine was also stage-specific, as the concentrations were so low in the juvenile tissue samples that they could not be reliably quantified. Alanine was the only FAA that was significantly higher in the juvenile samples relative to the larval samples, although this trend was restricted to the mantle and adductor tissues.

### 2.2. Metabolite Concentrations during Hypoosmotic Expsoure

There was a general trend of decreasing concentration for the five predominant amino acids—taurine, betaine, glycine, homarine, and alanine—in the gill, mantle, and adductor muscle of juvenile *M. edulis* when the mussels were exposed to low salinity treatment (Table 2). In both the gill and mantle tissues we detected significant multivariate main effect of salinity treatment on the variation in concentration of these five amino acids (gill: Wilk’s λ = 0.111, F_15,33.52_ = 2.717, *p* = 0.008, power = 0.931; mantle: Wilk’s λ = 0.163, F_15,33.52_ = 2.080, *p* = 0.039, power = 0.832). The changes were driven by a 72% and 35% drop in glycine concentration over the first 24 h of exposure in the gill and mantle, respectively. However, there was no evidence of univariate effects of salinity treatment on individual amino acids after adjusting *p*-value for the multiple one-way ANOVAs (critical value *p* < 0.01). In contrast, we did not detect a significant multivariate main effect of salinity on FAA concentrations in the adductor muscle (Wilk’s λ = 0.268, F_15,33.52_ = 1.368, *p* = 0.219, power = 0.612). 

Veliger and pediveliger larvae that were exposed to low salinity decreased the concentrations of six osmolytes at all time points tested (Table 3). In addition to taurine, betaine, homarine, glycine, and alanine, we monitored changes in the concentration of β-alanine in larval mussels, which was more abundant in veliger and pediveliger mussels compared to juveniles (Figure 3). We detected significant multivariate main effect of salinity treatment on the concentration of these six amino acids (Wilk’s λ = 0.015, F_18,25.94_ = 4.941, *p* < 0.001, power = 0.999). Examination of univariate effects of salinity treatment determined a significant effect of treatment on the concentration of glycine (F_3,14_ = 25.97, *p* < 0.001, power = 1.00) after adjusting *p*-value for the multiple one-way ANOVAs (critical value *p* < 0.008). Similarly, we detected significant multivariate main effect of salinity treatment on all six amino acids (Wilk’s λ = 0.012, F_18,20.24_ = 4.257, *p* = 0.001, power = 0.998). Examination of univariate effects of treatment determined a significant effect of treatment on the concentration of glycine (F_3,12_ = 25.97, *p* < 0.001, power = 0.993) after adjusting *p*-value for the multiple one-way ANOVAs (critical value *p* < 0.008).

## 3. Discussion

A primary goal of our project was to document stage- and tissue-specific differences in the abundance of free amino acids (FAA). Marine bivalves are osmoconformers that rely on selective adjustment of the concentration of osmolytes, especially free amino acids, when confronted with hyperosmotic or hypoosmotic conditions [15,16,17,18]. In addition to functioning as osmolytes, FAAs act to maintain a stable intracellular environment [30] and serve as stores for protein synthesis or energy metabolism [18]. While numerous studies have investigated changes in the FAA pools for *M. edulis* and other shellfish in response to osmotic stress e.g., [19,21,22], few have considered how the metabolome responds at different ontogenetic stages. We capitalized on the power and utility of ^1^H NMR to document ontogenetic variation in the metabolomic signatures, with a focus on FAAs, for mussels reared under ambient conditions, as well as in the response of the mussels’ metabolome during exposure to hypoosmotic conditions. 

### 3.1. Baseline Metabolic Profiles

Our ^1^H NMR-based metabolite study in *Mytilus edulis* resolved 99 metabolites in two larval stages and three tissues sampled from juvenile mussels. Overall, we identified 22 of these metabolites (Table 1), which is comparable to what has been identified in other metabolomic studies of blue mussels [3,8,9,29]. We observed striking differences in the metabolic signatures across developmental stages and among tissues of post-metamorphic mussels acclimated to ambient conditions (13.5 °C, 32 ppt). Of the metabolites detected in our study, only 9 were common among both larval samples and within the three tissues we sampled from juvenile blue mussels (Table 1). An additional 10 metabolites were present in larval and juvenile mussels, but were not detected in one or more stages or tissues. However, the prevalence of these metabolites (many of which are organic osmolytes) among tissues and throughout development reveals their importance to cellular function in *M. edulis*. 

In juvenile mussels, we found that taurine and betaine were the most prevalent organic osmolytes found, regardless of tissue type (Figure 3). Overall, the concentration of taurine in the tissues we examined was similar to that observed in previous studies of the adductor muscle [19,31] and digestive gland [21]. However, the concentration was 50% higher in gill compared to mantle tissue and 80% higher than in adductor muscle, although these differences were not statistically significant. Taurine is often cited as the most abundant osmolyte and constitutes 28–83% of the FAA pool in blue mussels [15,21,32,33,34]; however, betaine was not measured in those studies. Our findings suggest that taurine and betaine are important osmolytes in *M. edulis* and that previous studies, such as those of Wright et al. [32] and Deaton [35], may have overestimated the contribution of taurine and underestimated the contribution of betaine to FAA pools. Cappello et al. [27] similarly reported that the intensity of the peaks for taurine and betaine, along with glycine, were upwards of 100-fold higher than other metabolites in the gill and adductor muscle tissues, respectively, of *M. galloprovincialis*. Taurine and betaine act as counterbalancing solutes and promote cellular stability [16,36], so relatively high concentrations of these metabolites are important to the maintenance of cellular function in both stressed and unstressed mussels. Furthermore, these solutes are not synthesized by post-metamorphic mussels and must be taken up from the environment or obtained through the diet [37], so retention at high concentrations is likely to be energetically advantageous and osmotically important.

Glycine is also a common osmolyte found in mussels. High concentrations of this metabolite do not affect enzyme stability [16,17], it is naturally abundant in seawater [38,39,40], and it is synthesized via multiple metabolic pathways [24]. We observed significant differences in the concentration of glycine among three tissues of juvenile mussels (Figure 3). Glycine concentrations were lowest in mantle and significantly higher in adductor relative to the other two tissue types we examined. These tissue-specific patterns of glycine abundance are comparable to those reported by Shumway [19], Wright et al. [33] and Zandee et al. [41]. Tukinov et al. [26] and Ellis et al. [9] also reported differences in the amount of glycine among tissues in oysters and in the mantle of male and female *M. edulis*, respectively, which both authors attributed to glycine’s role in energy metabolism via oxidation or conversion to pyruvate. Overall, variation in glycine content among the tissues of *M. edulis* is likely a reflection of differences in the aerobic, metabolic demands of each tissue.

Other prominent FAAs we observed include alanine, homarine, and β-alanine. The concentration of alanine in adductor and mantle tissue was approximately 2-fold higher than in the gill (Figure 3). Although Wright et al. [33] did not find any differences in the alanine concentrations in the gill and mantle of *M. edulis*, Rice and Stephens [32] observed that alanine concentrations in the gill were 10-fold higher than the mantle and 50-fold higher than in the adductor, which is the opposite of what we found. Combined, these results suggest that concentrations of alanine are likely highly variable and context-specific and likely reflect tissue-specific metabolism. *M. edulis* larvae are also capable of taking up alanine rapidly from the environment and incorporating it into protein [24], so the low levels of alanine we observed in our larval samples may reflect the transfer of alanine out of the FAA pool into proteins.

We also detected homarine (N-methyl picolinic acid), a compound that is typically considered a metabolic byproduct in marine invertebrates [42]. Although not commonly studied in bivalves, studies in crustaceans suggest that homarine is derived from glycine and may be involved in methylation reactions to create betaine [43] or may itself function as an osmolyte [44]. The concentrations of homarine were highest in the adductor muscle and both larval stages, and lowest in the gill (Figure 3). Given the gill is one of the first tissues to respond to osmotic stress [45], our observation suggests that homarine does not play a leading role in osmoregulation. Instead, because glycine concentrations were also high in the adductor, we propose that homarine variation reflects differences in betaine metabolism among the tissues we sampled. 

In bivalve larvae, previous studies suggest that taurine constitutes approximately 70% of the FAA pool [46,47]. In contrast, we found that taurine only accounts for approximately 40% of the FAA concentrations in veliger and pediveliger larvae held under ambient conditions and the concentration of taurine was only 30% higher than those of betaine and glycine. These results suggest that taurine abundance is lower than previously reported while glycine and betaine are found in higher abundance. There were also stage-specific differences in the abundance of FAAs, as the concentrations of taurine and betaine were notably higher in the pediveligers than in the veligers or juvenile tissues (Figure 3). We attribute this to higher rates of taurine biosynthesis at this stage, because larvae, unlike post-metamorphic mussels, obtain taurine through de novo synthesis [25]. Glycine concentrations in the larval samples were over 40% higher than what has reported for any of the tissues of the juvenile mussels (Figure 3); glycine together with taurine and betaine accounted for over 80% of the total FAA we quantified in larvae. Manahan [24] reported that the uptake of glycine in bivalve larvae was faster than in juveniles. Considering the metabolic demands for growth in veliger and pediveliger larvae, it is perhaps advantageous for larvae to retain high intracellular levels of glycine. 

Interestingly, we observed appreciable concentrations of β-alanine only in mussel larvae. β-alanine can act to stabilize proteins [16], is important for redox balance [37], and often serves as an osmolyte in a variety of marine invertebrates [37,48]. Given these functions, it is surprising that we did not detect β-alanine in the juvenile samples. Livingstone et al. [21] reported that β-alanine made up only 0.3% of the total FAA pool in the digestive gland of *M. edulis*, while in larvae, we found it was more abundant and accounted for more than 7% of the metabolites quantified. The presence of β-alanine is shown to block uptake of taurine in the gill of *M. californianus* [23]. Thus, we interpret the higher concentrations of β-alanine in larvae as further evidence of high levels of taurine synthesis, rather than uptake, in the larval stages.

### 3.2. Metabolite Concentrations During Hypoosmotic Exposure

Glycine accounted for the largest change in the concentration of FAAs during hypoosmotic exposure in both larval and juvenile mussels. Overall, we found evidence for significant changes in FAA concentrations in gill and mantle tissues but not in the adductor muscle of juvenile *M. edulis* during a 72 h exposure to low salinity. The adductor muscle responds differently compared to the gill and mantle tissue with respect to variation in the concentrations of glycine, betaine, and taurine. These tissues vary in their permeability [33], in the number of solute transporters found within each of the tissues [32], and in the indirect effects of osmolytes on cellular function [37]. Glycine concentrations, in particular, decreased by 2–3 fold in these tissues, although these differences were not statistically significant when accounting for multiple comparisons. Glycine is considered an important osmolyte, however, Kluytmans et al. [49] has shown that its concentration varies seasonally in blue mussels depending on cellular energy demands. Given the prevalence of glycine in the cell, along with low energy content, its loss during hypoosmotic stress is likely relatively energetically inexpensive. 

The role of taurine during the salinity response in blue mussels has received much more attention than has glycine [19,21,22,31]. We did not detect any substantive change in the concentrations of taurine or betaine among any of the tissues we sampled from low salinity challenged juvenile mussels. This contrasts with the study of Bricteux-Grégoire et al. [31] who observed decreases in betaine and taurine in the adductor muscle after 48 or 72 h of exposure to low salinity. Other authors have also reported changes in the intracellular concentrations of taurine in *M. edulis* but only after prolonged exposure (weeks) to low salinity [21,50,51]. Losses of taurine and betaine are likely energetically expensive and both metabolites may provide a benefit to the cell during osmotic stress [16,52], so loss of these osmolytes would only be expected during longer exposures when the mussel is experiencing higher levels of stress.

This is the first study to evaluate changes in the FAA pools of larval mussels under hypoosmotic conditions. As with the juvenile mussels, we detected significant effects of low salinity exposure on FAA concentrations in veliger and pediveliger larvae. Glycine content began to decrease within 6 h of exposure (data not shown); this decrease was statistically significant and persisted through 72 h (Table 2 and Table 3). The magnitude of the glycine flux in larvae was much larger than what we observed in juvenile mussels. In salinity-treated veligers and pediveligers, there was a roughly 70% decrease in glycine compared to its 50% decrease in the gill and mantle tissues of juvenile mussels. Larvae have higher rates of leakage than juvenile mussels because of their increased surface area to volume ratio [24], which increases the likelihood of FAA loss to the environment relative to juveniles. Larvae also expend considerable amounts of energy on growth [14], which may be accounted for by increased energy metabolism though glycine [49]. 

Although not statistically significant after adjusting for multiple comparisons, there were also appreciable decreases in the concentration of β-alanine in larval mussels during low salinity exposure (Table 3). The concentration of β-alanine was 50% lower in veligers and 73% lower in pediveligers after 72 h exposure to low salinity. In bivalves, β-alanine is synthesized from aspartate or from the polyamine spermine [53] making it relatively easy to replace following low salinity exposure. As discussed above, we did not observe appreciable concentrations in β-alanine in juvenile mussels, suggesting that if β-alanine plays a role in salinity response, this response is unique to larval mussels.

Taurine and betaine concentrations were also decreased among larvae in treatment versus control groups. In larval bivalves, taurine is synthesized de novo [25], but in post-metamorphic mussels it must be obtained from the diet [37,54]. Thus, it is surprising from an energetic standpoint that larvae would have greater decreases in taurine relative to juveniles. However, the dietary need for taurine increases with age in *C. gigas* larvae [25], so it is possible that increases in metabolism during stress contribute to a greater taurine loss in larvae. Relative to juvenile mussels, the high energy demands of larvae may lead to increased susceptibility to hypoosmotic stress.

To our knowledge, this is the first NMR-based metabolomic study to examine the composition of FAA pools in blue mussel larvae. We observed numerous metabolites that were unique to veliger and pediveliger larvae, including two unknown metabolites at 2.92 and 2.96 ppm (see Figure 2). The intensity of these peaks was comparable to that of glycine or homarine, indicating these are prominent components of the mytilid larval metabolome. Unfortunately, both peaks had a single resonance and did not appear to have any coupling partners, so determining their identity using online databases was not possible. As veligers, mussels invest substantial amounts of energy into tissue growth and deposition of the larval shell (prodissochonch II [13]), so it is likely that many of these metabolites are byproducts of energy metabolism or shell secretion. Pediveligers, on the other hand, are preparing for metamorphosis [13], are rapidly increasing in size [47], and have developed a pedal organ. The unidentified solutes found with pediveliger may reflect metabolic differences associated with the developmental maturation, tissue growth, or the abundance of neurotransmitters.

## 4. Materials and Methods 

### 4.1. Sample Collection

The larval *Mytilus edulis* used in this study were cultured at the Darling Marine Center Hatchery in Walpole, ME, at an ambient temperature and salinity (13.5 °C, 32 ppt). Briefly, adult mussels were induced to spawn in June 2015 by exposure to cyclic thermal shock (14–21 °C), and their gametes were used to create five genetically-distinct replicate pools of larvae (2 males and 4 females per pool). The larval cultures were fed daily a mixture of *Monochrysis* sp., *Chaetoceros muelleri, C. calcitrans, Tetraselmis chuii,* and *Isochrysis galbana* (clone T-Iso), per the recommendations for bivalve aquaculture [55]. At 14 d post-fertilization (dpf), when the larvae had developed to the veliger stage, a subsample of approximately 100,000 larvae was removed from each replicate tank and placed into a 1 L beaker containing low salinity (20 ppt) UV-sterilized, filtered seawater (UV-FSW; 13.5 °C) and held 24, 48, or 72 h (*n* = 5 replicates, each time point). Another subsample of larvae from each replicate tank were held in control conditions (32 ppt UV-FSW; 13.5 °C) for 24 h. We conducted water changes every 24 h and starved the larvae for the 24 h prior to sampling. At the end of each treatment, the larvae were isolated on a 48-µm sieve, transferred to a sterile 1.5 mL Eppendorf tube, flash frozen in liquid N_2,_ and stored at −80 °C until analysis. At 26 dpf when the remaining larvae had transitioned to the pediveliger stage, another subsample containing approximately 100,000 pediveligers was placed into 1 l beakers containing low salinity (20 ppt) UV-FSW (13.5 °C) and held for 24, 48, or 72 h. We also held a subsample from each replicate tank at 24 h in control conditions (32 ppt UV-FSW; 13.5 °C). As before, the pediveligers were sieved, flash frozen, and stored at −80 °C. Unfortunately, prior to 26 dpf, there was high mortality in one of the larval replicate tanks so only four replicates were taken at the pediveliger stage. 

Juvenile *M. edulis* (26–45 mm in length) were collected from a subtidal population at the Darling Marine Center and transported to the University of Maine, Orono, ME, in September 2015. The mussels were held in a recirculating tank containing artificial seawater (Crystal Sea^®^, Marine Enterprises, LLC, Baltimore, MD, USA) and fed a daily ration of Shellfish Diet 1800 (Reed Maricutlure, Inc. Campbell, CA, USA). Following a 3-week acclimation to 15 °C and 32 ppt, the mussels were placed in separate 1 L beakers containing low salinity (20 ppt; 15 °C) artificial seawater (ASW) for 24, 48, or 72 h. As with the larval experiments, we exposed another set of mussels to control conditions (32 ppt ASW; 15 °C) for 24 h. We conducted daily water changes and all mussels were starved for 24 h prior to sampling for metabolic profiling. At the end of each exposure, juvenile mussels were sacrificed (*n* = 5) and the gill, mantle edge, and posterior adductor muscle from each were dissected out; the tissues were flash frozen in liquid N_2_ upon dissection and stored at −80 °C. To avoid gamete-specific differences in the metabolic signatures [6] for the mussels used in this study, we avoided using the gametogenic portion of mantle. The gonad of mussels forms in the visceral mass but during reproductive periods it extends significantly into the mantle [56], so we sampled tissues from young mussels that did not show signs of gonad production. 

### 4.2. NMR Spectroscopy

The larval and juvenile tissue samples were processed to examine the metabolic content of each sample using ^1^H nuclear magnetic resonance spectroscopy (NMR). A small section of each tissue or a subset of each larval sample was placed into a pre-weighed, sterile, 1.5 mL tube. The tissue was dried overnight at room temperature in a Savant™ SpeedVac™ Concentrator (Thermo Scientific™, Waltham, MA, USA). The dried samples were ground using a mortar and pestle and the dry weight of each sample was measured. Metabolites were extracted by adding 30 mL·g^−1^ dry weight of 2:1 acetonitrile–water mixture to the sample and vortexed to mix [57]. Samples were spun at 13,000 g for 10 m at 4 °C and the supernatant, containing the extracted metabolites, was removed and stored at −80 °C. We used 100 µL of the supernatant from each sample; each 100 µL sub-sample was dried in the SpeedVac™, resuspended in 500 µL deuterium (D_2_O), and dried again to replace residual water. Two D_2_O exchanges were conducted before the samples were redissolved in 500 µL D_2_O containing 2 mM trimethylsilylpropanoic acid (TSP) and 1.96 mM maleic acid, which were used as internal reference standards for relative quantification. The extracts were then transferred into Wilmad^®^ (Vineland, NJ, USA) 5 mm glass NMR tubes and stored at 4 °C until analysis. 

Samples were run on a 400 MHz Varian Inova NMR Spectrometer at room temperature. We acquired data using the instrument’s default parameters, modified to include a 5000 Hz spectral width, 13.5 µs pulse width, 1 Hz exponential line broadening, and no recycle delay. We acquired 64 transients for each spectra, which allowed us adequate resolution for quantification of the metabolites of interest. The transmitter was offset to the D_2_O peak and chemical shifts were referenced to TSP (0 ppm). Standards containing the amino acids betaine, glycine, taurine, proline, glutamate, and ornithine were also run under the same specifications to aid in the identification of common metabolites. 

A representative sample from each larval stage or tissue type was analyzed using 2D total correlation spectroscopy (TOCSY) to observe the coupling patterns of the components within each sample. These data provided structural information that helped verify the presence of amino acids and other metabolites in the corresponding 1D NMR data. The 2D spectra were obtained on the Varian 400 NMR Spectrometer at 30 °C. We acquired the 2D NMR using a 2.8 s recycle delay, a 30 µs pulse width, 1 Hz line broadening, and 64 transients over 512 t_1_ increments, for a total of 2048 t_2_ data points.

### 4.3. Data Analysis

The 1D NMR spectra were processed using ACD/NMR Processor Academic Edition Software. Data were Fourier transformed with a backward linear prediction for the first 2 points and then baseline corrected, phased, and calibrated to the TSP peak prior to analysis. For relative quantification, we manually selected and integrated the peaks for TSP, alanine, β-alanine, taurine, glycine, betaine, homarine, and maleic acid (Table 4). For each compound of interest, we calculated the concentration following the method of Bharti and Roy [58]:

(1) Mi =(IiHiIsHs)*Ms

where *M* is the relative concentration, *I* is the integral of the peak(s), and *H* is the number of protons contributing to the signal for the peak of interest (*i*) relative to the maleic acid standard (*s*). The concentrations for the metabolites of interest were then standardized to the mean TSP concentration and adjusted for dry weight, to yield the relative concentration in µmol g^−1^ dry tissue weight. 

To make direct comparisons of the metabolites quantified from the larval and juvenile samples, we adjusted the dry weights for the larval samples to account for the mass contributed by the larval shell. For this analysis, we prepared Whatman GF/C™ Glass Microfiber filters (25 mm diameter) by soaking in reverse osmosis (RO) water for 1 h and drying them overnight at 65 °C. The filters were then ashed at 350 °C overnight in a Thermo Isotemp^®^ (Waltham, MA, USA) Muffle Furnace and weighed. We placed a subset of larvae from each larval sample onto a filter and rinsed using 10 mL of 0.5 M ammonium formate to remove any residual salts from the seawater. The prepared samples were dried overnight at 65 °C and weighed (total dry weight), prior to ashing at 350 °C overnight to determine the ash-free dry weight (AFDW). The ratio of AFDW relative to dry weight was used to determine the proportion of the dry weight accounted for by the tissue (and not shell). The amino acid concentrations for each of the larval samples were then scaled by this proportion.

The 2D NMR spectra were processed using SpinWorks 3.1 NMR data processor. We Fourier transformed the data using complex forward linear prediction, predicting from 2048 to 4096 points in the F2 dimension and from 512 to 2048 points in the F1 dimension. Spectra were baseline corrected, phased, and calibrated to the TSP peak at 0 ppm. The coupling partners were recorded for each peak from the representative veliger, pediveliger, gill, mantle, and adductor samples and put into an Excel spreadsheet. The identification of metabolites was completed manually through reference to the primary literature and the Human Metabolome Database [59]. 

Tissue-specific or stage-specific variation in the relative concentration of the key metabolites alanine, taurine, glycine, betaine, β-alanine and homarine were determined by analyzing the solute concentrations for larval and juvenile mussels held under ambient, control conditions using ^1^H NMR spectroscopy. To test for tissue- and stage-specific differences in the relative quantities of these metabolites we ran a one-way MANOVA in SPSS Statistics 22.0 (IBM Corporation, Armonk, NY, USA), with sample type (juvenile tissue or larval stage) as a fixed-factor, main effect using a Type III Sum of Squares model with an experiment-wide α = 0.05. We examined the univariate effects of sample type on the concentration of individual amino acids using separate one-way ANOVAs with sample type as a main effect against a Type III Sum of Squares model and adjusting the critical value (*p* = 0.05/6 = 0.008) to account for multiple comparisons.

We investigated temporal variation for the five most abundant metabolites contributing to the FAA pool in juveniles under hypoosmotic conditions, glycine, alanine, taurine, betaine, and homarine. To test for the effects of low-salinity exposure on the concentrations of alanine, betaine, glycine, homarine, and taurine in juveniles, we used three one-way MANOVAs, one for each tissue type, with salinity treatment (control versus 24, 48, or 72 h exposure to 20 ppt) as a fixed-factor main effect using a Type III Sum of Squares model with an experiment-wide α = 0.05. We examined the univariate effects of salinity treatment on the concentration of individual amino acids using separate one-way ANOVAs with treatment as a main effect against a Type III Sum of Squares model and adjusting the critical value (*p* = 0.05/5 = 0.01) to account for multiple comparisons within each tissue type. Similarly, we investigated the temporal variation in six amino acids (those listed above plus β-alanine) in veliger and pediveliger larvae using two one-way MANOVAs. We examined the univariate effects of salinity treatment on the concentration of individual amino acids in larval stages using separate one-way ANOVAs with treatment as a main effect against a Type III Sum of Squares model and adjusting the critical value (*p* = 0.05/6 = 0.008) to account for multiple comparisons within each stage. Four cases were excluded from analysis because of sample loss during preparation.

## 5. Conclusions

This study contributes to our understanding of the metabolic baselines of veliger and pediveliger larvae and highlights important biochemical differences in the FAA pools of various tissues of post-metamorphic *M. edulis*. A number of studies have examined variation in the FAA pools of post-metamorphic *M. edulis* e.g., [19,21,22]; our inclusion of more tissue types and larval stages complements and builds on their foundation. We found variation in the composition and utilization of FAAs within larval and juvenile mussels, which provides insight into the role of these metabolites in energy metabolism and maintaining osmotic balance within the cell. Furthermore, many of the metabolites measured in this study function as osmolytes, so understanding their distribution and abundance across tissues and life-history improves our understanding on the capacity of different developmental stages to respond to salinity and other environmental stressors.

Variation in the baseline concentrations of these osmolytes among various tissues and developmental stages likely plays a role in the observed responses to hypoosmotic stress. Larval mussels—which vary from post-metamorphic mussels in form, function, lifestyle, and habitat—showed differences in their utilization of osmolytes during low salinity exposure. A better understanding of the metabolome of larvae, as well as their ability to regulate FAA pools provides insight into why larval mussels are more susceptible to hypoosmotic stress than juvenile or adult mussels [60]. Similarly, tissue-specific variations in the utilization of metabolites during low salinity treatment in juveniles reflected variation in the function of these tissues, which helps improve our understanding of the osmotic stress response in post-metamorphic mussels. Together, these observations highlight the importance of foundational metabolomic studies that include multiple tissue types and developmental stages to adequately evaluate organismal responses to stress and better place their findings in a broader ecological context.

## Figures and Tables

**Table 1:** Table of common metabolites identified in *Mytilus edulis.*

No	Metabolite	Chemical Shift (Multiplicity) ^1^
**Common to all samples**		
1	Alanine	1.46 (d), 3.77 (t)
2	Aspartate	2.68 (dd), 2.82 (dd), 3.87 (dd)
3	Betaine	3.26 (s), 3.91 (s)
4	Glycine	3.55 (s)
5	Homarine	4.36 (s), 7.96 (m), 8.03 (d), 8.53 (m), 8.69 (d)
6	Hypotaurine	2.63 (t), 3.36 (t)
7	Isoleucine	0.98 (m), 1.03 (m)
8	Taurine	3.25 (t), 3.43 (t)
9	Threonine	1.32 (d), 3.58 (d), 4.25 (t)
**Common to larvae and juveniles**		
10	Arginine	1.73 (m), 1.93 (m), 3.23 (m), 3.76 (m)
11	β-Alanine	2.56 (t), 3.18 (t)
12	Glutamate	2.05 (m), 2.14 (m), 2.38 (m), 3.76 (m)
13	Glutamine	2.14 (m), 2.42 (m), 3.76
14	Leucine	0.96 (t), 1.72 (m)
15	Lysine	1.73 (m), 1.88 (m), 3.02 (t)
16	Unknown #2 ^2^	1.24 (s)
17	Unknown Metabolite	0.87 (m)
18	Unknown Metabolite	3.66 (m), 4.28 (d)
19	Unknown Metabolite	3.75 (d), 4.27 (t)
**Larvae-specific**		
20	Unknown Metabolite	2.92 (s)
21	Unknown Metabolite	2.96 (s)
22	Lactic acid	1.32 (d), 4.12 (m)
**Juvenile-specific**		
23	Unknown #1 ^2^	1.09 (s)
24	Unknown Metabolite	2.25 (s)
25	Unknown Metabolite	2.56 (s)
26	Unknown Metabolite	3.11 (s), 3.28 (s)

^1^ The chemical shift (in ppm, referenced to TSP) and the type of peak are listed for each metabolite, where s = singlet, d = doublet, dd = doublet of doublets, t = triplet, and m = multiplet; ^2^ Metabolites listed by identity from [26].

**Table 2:** Free amino acid concentrations ^1^ in juvenile mussels following low salinity exposure.

Gill		Exposure at 20 ppt ^2^				
	Control	24 h	48 h	72 h	*F* ^3^	*p*
Taurine	357.3 ± 239.7	345.3 ± 36.6 (−3%)	343.2 ± 46.9 (−4%)	359.6 ± 45.0 (0%)	0.02	0.995
Betaine	266.2 ± 163.2	277.0 ± 50.3 (+4%)	275.3 ± 61.3 (+3%)	306.1 ± 45.2 (+15%)	0.171	0.914
Glycine	62.8 ± 45.4	17.6 ± 6.0 (−72%)	14.8 ± 5.5 (−76%)	22.7 ± 11.3 (−63%)	4.48	0.018
Homarine	17.4 ± 8.3	13.3 ± 3.9 (−23%)	7.7 ± 4.1 (−56%)	16.3 ± 4.0 (−6%)	3.21	0.051
Alanine	34.2 ± 30.3	22.2 ± 7.7 (−35%)	23.5 ± 7.8 (−31%)	24.0 ± 10.0 (−30%)	0.54	0.665
**Mantle**						
Taurine	238.2 ± 54.9	224.6 ± 27.7 (−6%)	200.3 ± 61.8 (−15%)	220.3 ± 45.1 (−8%)	0.51	0.680
Betaine	243.3 ± 30.2	243.0 ± 50.9 (0%)	266.2 ± 65.2 (+9%)	255.3 ± 72.9 (+5)	0.19	0.904
Glycine	81.5 ± 10.3	52.9 ± 13.5 (−35%)	37.5 ± 38.1 (−53%)	37.8 ± 23.9 (−54%)	3.70	0.034
Homarine	34.2 ± 7.0	29.6 ± 7.6 (−13%)	27.3 ± 13.7 (−20%)	39.0 ± 15.8 (+14%)	0.99	0.422
Alanine	80.4 ± 25.5	73.1 ± 23.6 (−9%)	58.8 ± 65.0 (−27%)	53.9 ± 53.0 (−33%)	0.37	0.778
**Adductor**						
Taurine	195.6 ± 38.1	216.3 ± 54.1 (+11%)	263.8 ± 36.3 (+35%)	198.1 ± 33.6 (+1%)	2.93	0.065
Betaine	151.7 ± 20.3	177.7 ± 77.7 (+17%)	213.7 ± 61.4 (+41%)	158.6 ± 29.3 (+5%)	1.39	0.281
Glycine	141.6 ± 51.7	137.4 ± 16.9 (−3%)	110.3 ± 36.7 (−22%)	100.7 ± 53.0 (−29%)	1.13	0.365
Homarine	53.4 ± 19.7	57.8 ± 22.5 (+8%)	48.3 ± 14.7 (−10%)	65.4 ± 40.6 (+22%)	0.38	0.768
Alanine	80.8 ± 24.3	66.1 ± 16.4 (−18%)	58.6 ± 24.9 (−27%)	63.3 ± 13.1 (−22%)	1.11	0.373

^1^ Concentrations measured in µmol·g^−1^ dry weight ± SE; ^2^ Values in parentheses indicate the proportional change in the concentration of each individual amino acid, where decreases are highlighted in red; ^3^ The F and *p*-values in the columns, at right, correspond to separate univariate analyses testing the effect of salinity treatment for each amino acid within the MANOVA for each tissue type. Given multiple comparisons the critical value for each univariate test is set at a = 0.01.

**Table 3:** Free amino acid concentrations ^1^ in larval mussels following low salinity exposure.

Veliger		Exposure at 20 ppt ^2^				
	Control	24 h	48 h	72 h	*F* ^3^	*p*
Taurine	349.5 ± 86.2	309.9 ± 27.4 (−11%)	244.1 ± 60.6 (−30%)	272.0 ± 65.1 (−22%)	2.10	0.146
Betaine	251.9 ± 60.6	207.8 ± 9.7 (−18%)	168.3 ± 47.0 (−33%)	205.0 ± 55.9 (−19%)	1.94	0.170
Glycine	248.4 ± 72.9	82.4 ± 11.3 (−67%)	61.6 ± 24.1 (−75%)	59.6 ± 19.1 (−76%)	25.97	*<0.001*
β-alanine	77.9 ± 40.7	38.7 ± 10.3 (−50%)	38.6 ± 13.6 (−50%)	44.0 ± 25.7 (−44%)	2.37	0.115
Homarine	75.2 ± 21.4	54.6 ± 5.2 (−27%)	48.6 ± 11.4 (−35%)	49.5 ± 13.0 (−34%)	3.30	0.052
Alanine	13.5 ± 4.5	12.8 ± 2.7 (−5%)	10.9 ± 5.9 (−19%)	11.9 ± 7.6 (−12%)	0.17	0.918
**Pediveliger**						
Taurine	537.5 ± 228.3	450.0 ± 207.0 (−16%)	365.3 ± 158.1 (−32%)	297.3 ± 51.0 (−45%)	1.45	0.227
Betaine	408.7 ± 174.2	307.3 ± 136.4 (−25%)	249.2 ± 91.1 (−39%)	188.2 ± 15.9 (−54%)	2.45	0.114
Glycine	384.6 ± 167.9	122.9 ± 60.8 (−68%)	72.1 ± 22.8 (−81%)	48.5 ± 5.3 (−87%)	11.83	*0.001*
β-alanine	91.1 ± 46.6	54.1 ± 38.1 (−41%)	29.6 ± 21.6 (−68%)	25.0 ± 2.8 (−73%)	3.58	0.047
Homarine	70.0 ± 23.6	57.8 ± 25.0 (−17%)	49.4 ± 18.4 (−29%)	32.0 ± 4.2 (−54%)	2.64	0.097
Alanine	29.3 ± 18.6	18.7 ± 16.5 (−36%)	16.5 ± 3.9 (−44%)	9.5 ± 3.3 (−68%)	1.67	0.227

^1^ Concentrations measured in µmol·g^−1^ dry weight ± SE; ^2^ Values in parentheses indicate the proportional change in the concentration of each individual amino acid, where decreases are highlighted in red; ^3^ The F and *p*-values in the columns, at right, correspond to separate univariate analyses testing the effect of salinity treatment for each amino acid within the MANOVA for each tissue type. Given multiple comparisons the critical value for each univariate test is set at a = 0.01.

**Table 4:** Chemical shifts key osmolytes used for relative quantification.

Compound	Shift (ppm)	Type ^1^	H ^2^
TSP	0	s	9
Alanine	1.46	d	3
β-Alanine	2.54	t	2
Taurine	3.43	t	2
Glycine	3.55	s	2
Betaine	3.91	s	2
Homarine	4.36	s	2
Maleic Acid	6.32	s	2

^1^ s = singlet, d = doublet, and t = triplet; ^2^ the number of protons (H) contributing to the signal.

## Supplementary Materials

The following are available online at www.mdpi.com/2218-1989/7/3/33/s1. Table S1: Complete list of metabolites identified in *Mytilus edulis*.
