# Supplementary material for: NMR Profiling of Metabolites in Larval and Juvenile Blue Mussels (Mytilus edulis) under Ambient and Low Salinity Conditions
Source: Metabolites. 2017 Jul 6;7(3):33. doi: 10.3390/metabo7030033 (PMC5618318; doi:10.3390/metabo7030033)
Supplement: Supplementary file 1 [file metabolites-07-00033-s001.pdf]

Supplementary Materials: NMR Profiling of Metabolites in Larval and Juvenile Blue Mussels (*Mytilus edulis*) under Ambient and Low Salinity Conditions

Melissa A. May, Karl D. Bishop and Paul D. Rawson

Table S1. Complete list of metabolites identified in *Mytilus edulis*

No	Chemical Shift (ppm)	Possible Identity
**Veliger-specific metabolites**		
27	1.33, 1.57	
28	1.73, 4.18	
29	2.14, 2.23	
30	2.70, 3.58	Sarcosine
31	2.74, 3.27, 3.44	
32	2.97, 4.30	
33	3.02, 4.19	
34	3.09, 3.57	
35	4.13, 4.18	
36	4.36, 5.97, 7.95	
37	4.39, 4.49, 6.13	
38	4.43, 6.68	
**Pediveliger-specific metabolites**		
39	1.4 (d)	
40	1.78, 3.12	
41	2.22, 2.24	
42	2.72, 3.46	
43	3.60, 5.39	
44	3.61, 3.84	
45	3.63, 3.96	
46	3.75, 4.65	
47	3.90, 4.10	
48	4.17, 4.28	
49	4.27, 4.58	
50	5.32, 7.03	
51	7.85 (m)	
**Other larval-specific metabolites**		
52	3.37, 4.44	

53	3.79, 3.90	
54	1.86, 3.70	
55	2.35 (m)	
56	3.23, 4.65	
57	3.38, 3.43	
58	3.41, 3.69	
59	3.42, 3.80	
60	3.46, 3.71	
61	3.46, 4.64	
62	3.52, 3.70, 3.82, 5.22	Glucose
63	3.69, 3.72	
64	4.48, 5.13	
65	6.90	
66	7.19 (m)	
**Mantle-specific metabolites**		
67	1.47, 1.90	
68	3.07, 3.19	
69	3.11, 3.41	

Table S1, continued

**No**	**Chemical Shift (ppm)**	**Possible Identity**
**Mantle-specific metabolites, cont.**		
70	3.17	
71	3.34, 4.02	
72	3.48 (dd), 3.68 (dd)	Cysteine-S-sulfate
73	3.90, 3.96	
74	5.23, 5.30	
75	7.31	
**Adductor-specific metabolites**		
76	0.95 (m)	
77	1.08, 1.13	
78	1.19	
79	1.49, 3.70	
80	2.46, 3.41	
81	2.68, 2.73	Citric acid
82	2.76, 2.81	
83	2.86, 3.44, 3.59, 3.80	Unknown #3[1]
84	3.13, 3.24	
85	3.18, 3.22	
86	3.23, 3.75	
87	3.9 (m)	

**No**	**Chemical Shift (ppm)**	**Possible Identity**	**Source of Metabolite[2]**

**Juvenile-specific metabolites**			
88	2.25, 3.88, 4.12, 4.64		M, A
89	6.59		G, M
90	7.39 (m)		G, M
**Other metabolites**			
91	5.21 (d)	α-glucose	P, A
92	5.38 (d)	Allantoin	P, A
93	6.54 (s)		P, A
94	3.08, 3.41		P, G
95	4.11, 4.38		P, G
96	4.49, 6.14		P, G
97	3.06 (m)		P, G, M
98	8.29 (m)		P, G, A
99	8.33 (m)		P, G, M

[1]Metabolites listed by identity from [21]; [2]The source of metabolite is the tissue or stage that the metabolite was identified in, where A = adductor, M = mantle, G = gill, P = pediveliger, and V = veliger.
